# Microbeads and microcapsules for diet delivery to *Zelus renardii*

**DOI:** 10.1371/journal.pone.0334859

**Published:** 2025-10-17

**Authors:** Ugo Picciotti, Giuseppe Francesco Racaniello, Marianna Ivone, Pasquale Trotti, Angela Assunta Lopedota, Paolo Damiani, Francesca Garganese, Nunzio Denora, Francesco Porcelli

**Affiliations:** 1 Department of Soil Sciences, Plants and Food, University of Bari Aldo Moro, Bari, Italy; 2 Department of Pharmacy – Pharmaceutical Sciences, University of Bari Aldo Moro, Bari, Italy; Chinese University of Hong Kong, HONG KONG

## Abstract

Predation on Aphrophoridae and other olive tree pests makes *Zelus renardii* a candidate for biocontrol actions to limit *Xylella fastidiosa* infections while mitigating other olive tree pests. The opportunity drives the search for an effective mass rearing method of *Z. renardii*. Predator rearing on artificial diets greatly benefits from feed-effective formulation, preparation, storage, preservation, and delivery. Given the several oligidic, meridic, and holidic available formulations, we face the challenge of a proper diet processing for delivery. To understand how to obtain a large number of preservable and sterile diet portions while avoiding microbial contamination, we explore prilling/vibration techniques to rear *Z. renardii*. Prilling or vibrating the diets yields multicore microbeads or monocore microcapsules; water domains exist, whose arrangements are well-documented by the cryo-SEM study and represented in corresponding false-color images. Issues include the density interplay between low- or high-density alginate and the liquid diet formulation during prilling/vibration. Other options relate to alginate stickiness or consistency, which makes it difficult to disperse the diet domains in the microbeads or to obtain a single diet domain per microcapsule because of unpredictable wall thickness and core lateralization. We suggest options to make microbeads and microcapsule portions available for up to one year for predators, stored in cold, pure water.

## 1. Introduction

*Zelus renardii* Kolenati, 1856 (Leafhopper Assassin Bug, LAB) is a Reduviidae predator (Hemiptera: Harpactorinae) native to the Nearctic region [1,2] now established in Europe [3,4], and has also entered various countries in South America and Oceania [5–10]. The reduviid prefers hemipteran prey [11], suggesting a potential role in pest biocontrol [11–13]. Among its possible targets, *Philaenus spumarius* (L., 1758) (Hemiptera: Aphrophoridae) stands out as a known vector of *Xylella fastidiosa* Wells *et al*., 1987 (XF) subsp. *pauca* ST53 in southern Italy [14,15].

LAB predation on Aphrophoridae vectors could help reduce new infections, thereby mitigating XF invasion [13]. In addition, LAB can complement the IPM (Integrated Pest Management) of olive orchards to minimize damage by other olive pests, such as *Bactrocera oleae* (Rossi, 1790) (Diptera: Tephritidae) [11]. Therefore, the use of LAB as an inundative biocontrol agent depends on whether it can be mass reared.

Insect rearing is an ancient human need, first leading to the domestication of *Bombyx mori* L., 1758, for silk production between 5,000 and 4,100 years ago [16–18], likely originating from the wild ancestor of the silkworm, *Bombyx mandarina* Moore, 1872 (Lepidoptera: Bombycidae). Many other insects are reared or bred today [19]. Still, none have reached the level of domestication of silkworms.

The need for sustainable agri-food production and restrictions on synthetic chemical formulations or pest management [20] suggest the availability of robust alternative control actions [21]. Biocontrol offers viable and effective alternatives, relying on natural antagonists, such as predators or parasitoids [22], which are now commercially available for approximately 350 insect species [23].

International policies promote inundative and inoculative (augmentative) biocontrol to minimize pesticide use [24–26]. Several natural enemies are commercially available for pest management across various cropping systems [27,28]. Augmentative biocontrol applies through the periodic release of indigenous or alien mass-reared antagonists, which are suitable for the target agroecosystem [24]. Species from the families Reduviidae, Anthocoridae, Miridae, Geocoridae, Nabidae, and Pentatomidae work in augmentative biocontrol programs across various agroecosystems [29]. The efficacy and overall costs of augmentative biological control programs should match those of synthetic chemical control [26].

The mass-rearing of biocontrol agents facilitates field release, producing natural enemies in large quantities and proper quality [30]. Typically, the limitations of mass-rearing programs stem from the need to rear the natural enemies on their target or alternate prey, which is the most frequent choice for several ecto- and endoparasitoids (e.g., *Trichogramma* spp.) and some predators (e.g., *Chrysoperla* spp.) [31].

The costs and labor involved in rearing natural prey or hosts complicate the mass-breeding of many antagonists [32]. Furthermore, breeding antagonists on prey is not recommended due to the risk of devastating epizootic events caused by prey-driven diseases or other disturbances [31]. Therefore, rearing natural enemies on artificial diets may help to avoid these inconveniences.

Artificial diets must maintain their chemical and physical properties during storage, thereby facilitating better management of the reared antagonists’ feeding, health, and growth. The breeding of biocontrol agents on artificial diets began with Bogdanow [33], who first developed artificial diets for insects, paving the way for modern formulations. The *pabulum* must satisfy the species’ nutritional requirements for consistent and balanced development [34–37], enabling active and voracious antagonists suitable for field use. The compositions of artificial diets range from chemically defined (holidic) to semi-defined (meridic) to undefined (oligidic) [38,39].

There is a trend toward formulating diets that vary in components and doses, and designing diets that utilize predictive mixtures of ingredients based on the specific needs of insects [40]. Artificial diets also represent a breakthrough in enhancing rearing automation in antagonists mass production. Additionally, the use of predictive mixtures helps overcome issues for insect physiopathology [41].

The literature reports diverse solutions for diet delivery, depending on the species: packets of liquid diet [42,43], artificial feeders dispensing liquid diet for various insect species [44], or devices that dispense agar-gelled diets [45]. Biocontrol strategies based on biological augmentation still face challenges in developing mass-rearing programs [46], particularly in automating the rearing and mass production of antagonists.

Optimizing the artificial diet and its delivery is crucial for mass-rearing natural enemies, as it affects the quality and efficacy of feeding and, consequently, the success of the biological control agent and program [47]. The growing commercial demand for mass biological control agents in integrated agricultural production has heightened the need for effective artificial diets, adequate insect nutrition, rearing efficacy, ease of storage, and feeding capabilities [48,49].

The formulation of artificial diets often involves gelling agents to ensure an even distribution of diet sources [46]. Agar is the most widely used and longest-standing gelling agent [50], but it is also costly [51]. Furthermore, agar requires diet heating, which can compromise the quality of selected ingredients in artificial diets, particularly heat-sensitive vitamins [50].

Diets that use agar-gelling are also susceptible to microbial contamination, typically due to opportunistic or entomopathogenic [52] species. Bacteria and fungi rapidly contaminate the diets, compromising diet palatability and quality [53]. Microorganisms also contaminate the diet by catabolites, further impacting the breeding and exposing insects to various stresses. Transferring feral antagonists often compromises the breeding habitat, because bacteria or fungi associated with ferals quickly contaminate diet portions. Temperature and relative humidity can manage microbial invasion in insect rearing, thereby helping to mitigate microbial contamination.

Alginate is a gelling agent derived from brown algae, specifically *Laminaria* spp. and *Ascophyllum* spp. (Phaeophyceae) [54], widely used in food processing as a thickener, emulsifier, or stabilizer, particularly in the dairy industry [55].

Compared to agar, alginate is less expensive and does not require heating to gel, thereby preserving nutrient stability and vitamin activity, and easily giving microbeads or microcapsules. Indeed, alginate easily binds with bivalent cations, such as Ca^2+^, to form stable hydrogels [56].

Alginate entered into insect diets for delivery in the last 60 years [57], and various artificial diets, including alginate, helped insect mass-rearing; however, none employed the encapsulation technique [47,58,59].

Encapsulation in alginate beads can also disperse entomopathogenic nematodes [60–62], spread inoculum of biocontrol agents [63,64], or help the distribution of Plant Growth-Promoting Rhizobacteria (PGPR) or fungi (PGPF) [65–67]. The literature mentions a few earlier instances of insect artificial diets encapsulation. Still, these attempts primarily use waxes and plastic polymers as inclusion media [68] to directly enhance the efficiency and stability of mass rearing [69].

Our study primarily focuses on evaluating the quality of diet delivery devices, specifically microbeads or microcapsules, using diets that have already maintained *Zelus renardii* rearing [39]. Here, we share innovations on alginate-based devices (microbeads or microcapsules) to deliver meridic and holidic diets appealing to the predator. We presume that the devices will confer on diets a long shelf-life and portioning options, suited to the rearing program. Furthermore, devices will help insect rearing, sanitation and cleaning, operators’ safety and comfort, mass rearing management, and ease automation in diet distribution. Breeding automation appears to be a relevant target in view of a feasible and cost-effective predator mass production.

## 2. Materials and methods

### 2.1. Insect source

The feral LABs originate from unmanaged economic *Citrus* spp. in the “Ernesto Quagliariello” Campus, University of Bari Aldo Moro (N 41°06’37”; E 16°52’58”). The harboured *Citrus* hosted a mixed infestation of *Aleurocanthus spiniferus* (Quaintance, 1903), *Aleurothrixus floccosus* Maskell, 1896, and *Lepidosaphes beckii* (Newman, 1869). *Zelus renardii* developed alone in Petri dishes, under laboratory season-varying conditions. The minimum temperature was 18˚C in winter and the maximum was 25˚C in summer, kept by air conditioning. Blot paper water-soaked flooring and the vented Petri dishes maintained a proper relative humidity (RH) as in [11,13,39].

### 2.2. Diets formulation

The holidic diet (D1), based on Meritene MOBILIS^®^ (Nestlé S.A., Vevey, Switzerland), includes carbohydrates, amino acids, lipids, minerals, and vitamins. The meridic artificial diet (D4) contains Meritene MOBILIS^®^, KCl, and OB (Oligidic Base), while the meridic diet comprises chemically undefined components [39]. Feral, non-sanitized *Z. renardii* were maintained in the laboratory on agar-gelled D1 and D4 in Petri dishes, with daily cleaning to minimize contamination [39], before offering the alginate devices.

### 2.3. Preparation of microparticles by the prilling/vibration technique

A B395 Pro Encapsulator (Büchi Labortechnik AG, Flawil, Switzerland) produced microbeads or microcapsules by prilling or vibration, breaking a laminar flux polymer solution into one-dimensional droplets through a vibrating nozzle. The droplets fall into a consolidation bath where they solidify into microparticles [70–72], creating a matrix system, the microbeads, or shell-core structures, the microcapsules.

### 2.4. Multicore systems or microbeads

Four different formulations, in Table 1 below, corresponded to different microbeads, all starting from the holidic (D1) and meridic (D4) diets (Table 1).

**Table 1:** Device composition.

Formulation code	Meritene MOBILIS^®^ (w/v %)	OB (w/v %)	Alginate low viscosity (SA_l_) (w/v %)	Alginate high viscosity (SA_h_) (w/v %)
S1	17.0	--	--	2.0
S2	17.0	--	2.0	--
S3	17.0	5.0	2.0	--
S4	17.0	3.3	2.0	--

The Ultraturrax T25N (Ika, Königswinter, Germany) homogenized the S1, S2, S3, and S4 ingredients in distilled water at 13,200 rpm for 5 minutes. SA_h_ or SA_l_ completed the mixtures at 30°C with magnetic stirring, to dissolve the polymer.

The different recipes (Table 1) streamed through a single 1,000 μm ⦰ nozzle thanks to a syringe pump. Process parameters adapt to the viscosity of the final mixture. The laminar flux of the liquid vibrated at 50 Hz, determining the number and size of the droplets, and 1,000 V p.d. prevented droplet coalescence, maintaining 25 centimeters between the nozzle and the consolidation bath. A volumetric flow rate of approximately 4 ml/min with amplitude 2 worked for S1 and S2. In comparison, a flow rate of 8 ml/min and an amplitude of 4 run for S3 and S4. The droplets rested in a 0.1 M CaCl_2_ solution to obtain F1, F2, F3, and F4 devices. The microbeads rested in the gelling solution for 15 min at 25°C, then rinsed twice with distilled water. Additionally, F2 microbeads floated in a dilute OB solution (1% w/v) to create the F2_bis_ formulation. The microbeads run immediately or rest in water at 4°C until needed.

### 2.5. Monocore system or microcapsules

Different polymeric solutions shaped the microcapsule’s shell and core. A 1.5% w/v solution of SA_h_ created the shell, while a 3.3% w/v solution of OB with 0.2% w/v SA_l_ filled the core. A 700 µm nozzle poured out the core matter, and a 900 µm nozzle the shell. Diets D1 and D4 (Table 2) gave F5, F6, F7, and F8 formulations with different process parameters.

**Table 2:** Parameters for the preparation of microcapsules by the prilling/vibration technique.

Formulation Code	Inner and outer flow rate (mL/min)	Frequency (Hz)	Electrode potential (V)
F5	12/20	1,100	1,000
F6	14/21	80	750
F7	15/19.3	50	1,000
F8	3.5/13.72	40	1,000

Each polymer droplet gelled in a 0.3 M CaCl_2_ solution while gently stirring. The microcapsules floated in the gelling solution for 15 min at 25°C, then rinsed twice with distilled water. The microcapsules run immediately or rest in water at 4°C until needed.

### 2.6. Microbeads and microcapsules evaluation

An inverted light microscope, Optech IB 4 (Optech Microscope Services Ltd, Thame, UK), equipped with an image analysis software (Capture 2.1, SS&C Blue Prism, Warrington, UK), measured the microbeads and microcapsules. We evaluated the mean diameter and the relative standard deviation of at least fifteen microparticles from each formulation that had a proper diameter (Tables 3-4). Microparticles are relatively soft and subspherical with a wide range of sizes, and we repeated the measurement to minimize the error. We positioned each microparticle on a microscope slide and measured from left to right by sliding the micrometric table with the help of subjective and objective grids. We re-measured each particle using a digital sliding caliper (Hi-Tech Diamond Westmont, USA) to validate the measurements.

**Table 3:** Diameter of microbeads in water, and mean ± SD.

Formulation Code	Microbead diameter (mm)
F2_bis_	3.87 ± 0.28
F4	3.65 ± 0.15

**Table 4:** Diameter of microbeads. Data report the mean ± SD.

Formulation Code	Microbead diameter (mm)
F5	1.89 ± 0.12
F6	2.45 ± 0.14
F7	2.91 ± 0.23
F8	3.78 ± 0.28

Additionally, microbeads and microcapsules were frozen-split and imaged [73] using a cryo-SEM (Hitachi TM-3000, Hitachi High-Technologies Corporation, Tokyo, Japan) to study the internal structure of the matrix and the distribution domains within.

## 3. Results and discussions

### 3.1. Contamination

**Fig 1:**
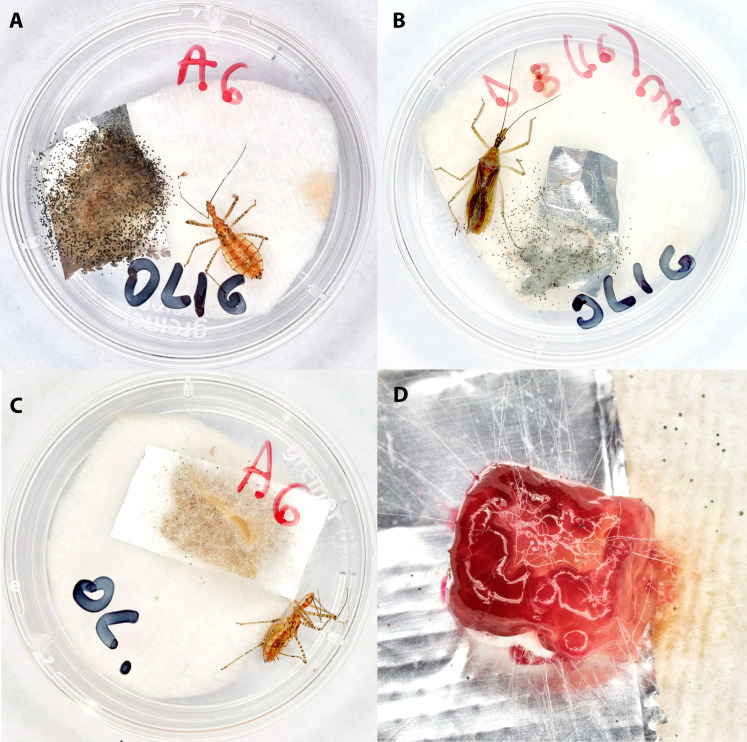
Nymph of feral *Zelus renardii* in a Petri dish with D4  + agar contaminated by *Aspergillus niger* (A); feral adult female of *Z. renardii* in a Petri dish with D1 + agar contaminated by *Rhizopus* spp. (B). Same nymph as in picture 1A, with another piece of D4 + agar contaminated again by *Aspergillus niger* (C); a piece of D4 + agar contaminated by bacteria, yeast, and fungi **(D)**.

**Fig 2:**
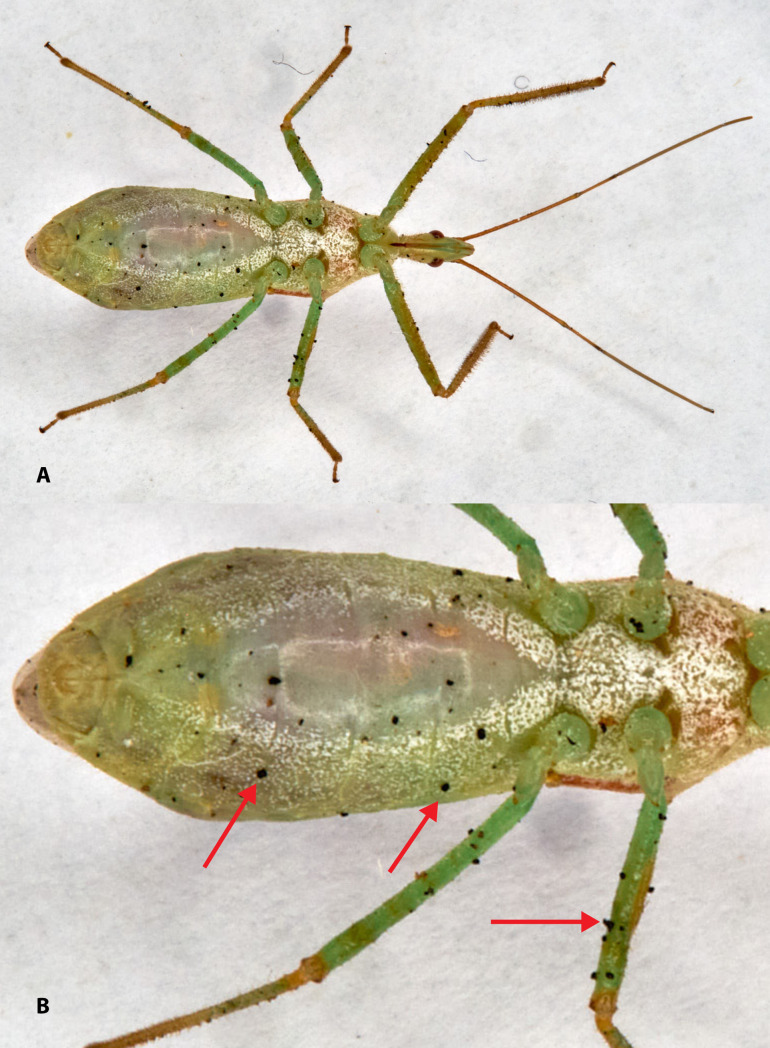
Ventral abdominal view of a wild female *Z. renardii* field-collected (A); enlarged, red arrows on dirty spots (B).

Frequent LAB-driven microorganism contaminations occur on diets during rearing experiences with feral *Z. renardii* (Figs 1 and 2). The most frequent fungal contaminants are *Aspergillus niger* Tiegh., 1867 (Fig 1A and C), *Aspergillus flavus* Link, 1809, *Penicillium* spp., *Rhizopus* spp*.* (Fig 1B), and yeasts (Fig 1D). Several contaminants have already been reported in the literature [74–77]. *Aspergillus niger* is the most frequent fungus found in our insect diet; its impact on the diet varies with the insect instar and the contamination interval [78].

Feral *Z. renardii* bear environment-collected microbial inoculum on their cuticle (Fig 2A and 2B), spreading the microorganisms. The sanitization of feral *Z. renardii* remains unexplored, as sanitizing agents can jeopardize the life of the reduviid.

Microorganism species, LAB instar/age, contamination interval, temperature, medium pH, and other factors apparently impact microbial contamination and, thus, the insect growth.

Management and contaminant mitigation or suppression are delicate tasks that must remain effective to reduce the microorganism population to a non-disturbing level [76]. Starting new rearing lines using wild eggs instead of postembryonic *Zelus* may minimize the contaminations otherwise frequently carried by free-living LAB. Operators’ hygiene, a clean rearing environment, commercially available sterile diet components, and media sterilization [79] may help maintain a reasonably clean rearing setup. Microbeads or microcapsules produced during this study remained stable for approximately one year in 4°C pure water. We presume that microbeads or microcapsules can maintain a diet free from contamination for a relatively long period.

### 3.2. Production and features of microbeads, the multicore device

**Fig 3:**
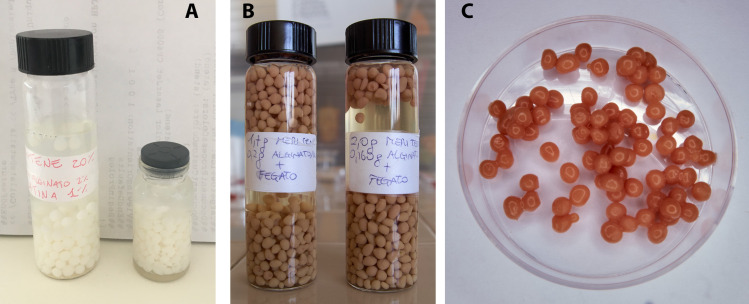
Vials with F2 (A) and F4 microbeads (B) immersed in pharmaceutical water; F4 microbeads drained (C).

Both diets were successfully portioned into microbeads using the prilling/vibration technique. The S1 and S2, which contained only Meritene MOBILIS^®^, differed in the SA viscosity. The S1, using SA_h_, had difficulty extruding through the nozzle due to its high viscosity. Consequently, SA_l_ was used in the S2 formulation, resulting in well-separated droplets and uniformly distributed microbeads (F2) (Fig 3A). Furthermore, F2_bis_ promotes a better distribution of the OB on microbeads and enhances insect palatability.

In S3 and S4, the amounts of Meritene MOBILIS^®^ and SA_l_ were constant compared to S2, but OB entered in varying concentrations. S3 caused partial nozzle clogging, resulting in a discontinuous droplet production process and inhomogeneity of the microbeads. Consequently, the amount of OB in S4 was uniform with a high production yield (F4) (Fig 3B and C).

The microbead diameters (F2_bis_ and F4; Table 3) were less than 4 mm.

**Fig 4:**
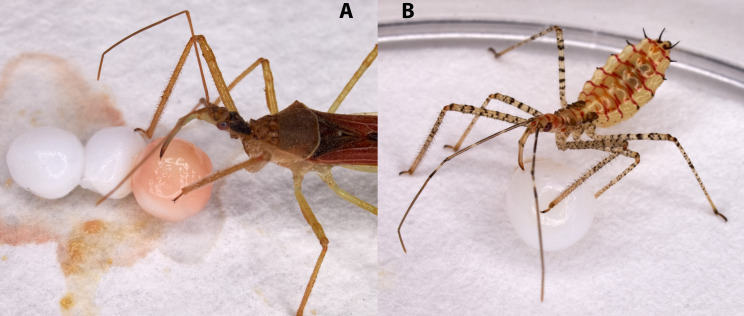
*Zelus renardii* probing on F2_bis_ (A, adult) and F2 (B, nymph).

The size of the microbeads aligns with the prey size preference for adult *Z. renardii*; therefore, the microbeads promoted feeding attempts by both adults and nymphs (Fig 4A and B).

### 3.3. Microbeads in section

**Fig 5:**
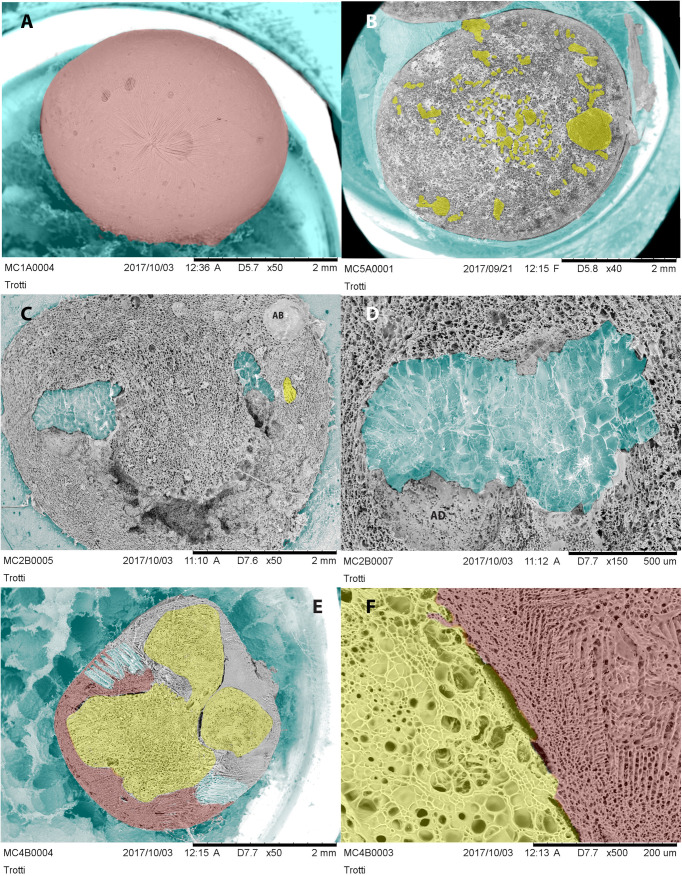
Cryo-SEM false color images of F2_bis_ (A), a cross-section of F2 (B), another cross-section of F2_bis_ with ice domains and an air bubble (C), details of ice domains in the F2_bis_ cross-section (D), F4 cross-section (E), and F4 separation interface between Meritene MOBILIS^®^ and OB (F). Legend: blue = ice domains; yellow = Meritene MOBILIS^®^ domains; red = oligidic base; white/grey = alginate matrix. Abbreviations: AB = air bubble; AD = alginate domains.

Figs 5A and 5B show the surface and the cross-section of F2_bis_ microbeads, respectively. Fig 5A shows that microbeads F2_bis_ maintain an Oligidic Base layer. However, the particle’s cross-section (Fig 5B) revealed a scattered distribution of Meritene MOBILIS^®^, resulting in several multicore dispersed small diet domains separated by consistent alginate walls. Fig 5C also shows that microbeads may have small Meritene MOBILIS^®^ domains separated into the alginate microbeads matrix. Furthermore, the multicore system incorporates several air domains, as shown as bubbles, AB, in Fig 5C. Small air bubbles, alginate walls, and uneven diet domains are technical issues that negatively impact the availability of the deliverable portions for the insect. Microbeads can also incorporate big water domains (Fig 5D) that could profitably serve as water reservoirs for predators during rearing. Fig 5E shows the cross-section of F4 microbeads, highlighting a clear separation between Meritene MOBILIS^®^ and OB, possibly due to the non-miscibility of the components at the time of prilling, giving an irregular multicore system (Fig 5F). F4 microbead shows a complex pattern of interplaying domains with volumes occupied by SA_l_ and OB in the outer layer and Meritene MOBILIS^®^ filling the microbead core (Fig 5E). OB and Meritene MOBILIS^®^ phases do not interact as the F4 cross-section highlights.

The cut surface of microbeads appears initially flat in cryo-SEM, revealing the domains and the distribution of components as a layer. The ice sublimation chisels the surfaces, revealing non-ice and non-sublimating matter. Freezing is fast, almost instantaneous, giving vitreous, non-crystalline ice. A core can disrupt the homogeneity of the alginate gel matrix, building domains, such as microchannels that run inside the gel particle [80].

The evidence shows that *Z. renardii* cannot effectively access and utilize microbeads as they are in this experiment. *Zelus renardii* attempts to feed on the microbeads, but the abdominal fold of the predator doesn’t stretch out, suggesting the attempt’s failure. In contrast, preying and feeding on living prey induces a fold stretch, demonstrating *Z. renardii’s* engorgement [11]. Moreover, the excessive dispersal of diet cores into small, isolated domains that are often inaccessible to the predator because they are unperceivable from the surface of the microbeads leads to an unfavorable trade-in/trade-off during feeding attempts. Therefore, these findings suggest the need to modify the microbead composition or the prilling/vibrating parameters appropriately.

### 3.4. Production and features of microcapsules, the monocore device

Microcapsule prilling/vibration aimed to achieve the monocore devices of about ≥3 mm, providing adequate diet portions to *Z. renardii*. We used several parameters for this purpose, with constant inner and outer nozzle diameters, but variable vibration frequency and inner/outer nozzle flow rates. The diameter of the microcapsules primarily depends on the outer nozzle diameter, with variations in size influenced by the flow rate and vibration frequency. The inner nozzle diameter and flow rate secondarily affect the microcapsule size; increasing these parameters results in larger core volumes and capsule sizes. Generally, higher frequencies and lower flow rates yield smaller sizes of the shell and core material [81].

Table 4 displays the diameters of microcapsules from various formulations (F5, F6, F7, and F8), indicating that a decrease in vibration frequency increases the microcapsule diameter. Formulation F8 fulfilled the requirements with an average diameter of 3.78 mm. The microcapsule cryo-SEM study shows the device’s internal structure.

### 3.5. Microcapsule in section

**Fig 6:**
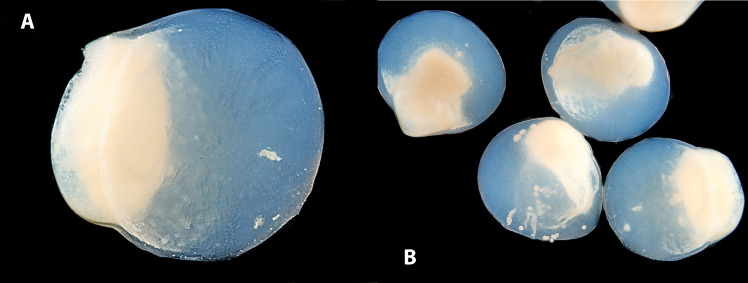
Lateral view of a single (A) or a group of F8 microcapsules (B) in light, dark field macroscopy.

Figs 6A and 6B, dark field and transmitted light, display the F8 microcapsules, which appear sub-spherical, with a decentralized diet core.

**Fig 7:**
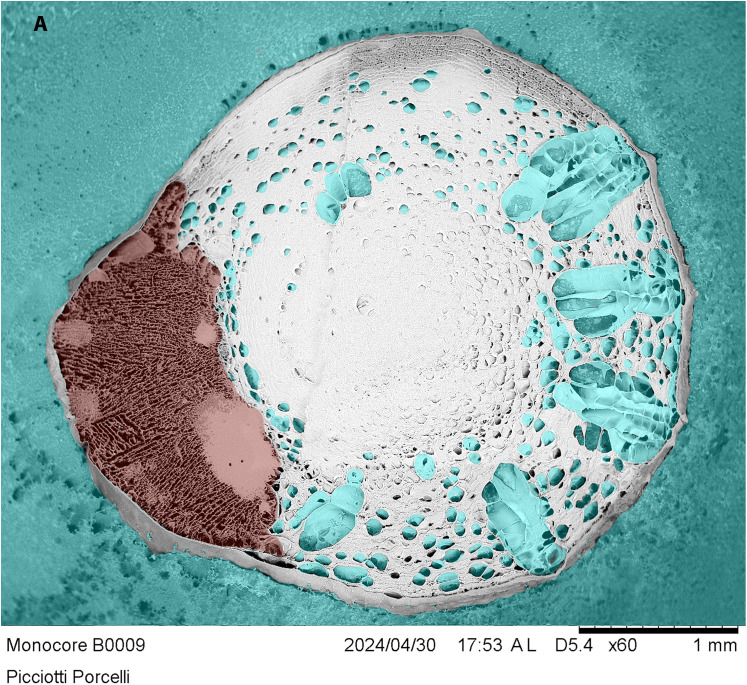
A cryo-SEM cross-section of the F8 microcapsule in Fig 3A. Legend: blue = ice domains; red = oligidic base; white/grey = alginate matrix.

The cross-section (Fig 7) reveals a single-core system with evident core decentralization, probably due to the excessive core-feed density. The microcapsule system features a thick wall on one side and an extremely thin alginate containment on the other. Differences in densities between OB and alginate may generate such a core-side shift. The OB core appears homogeneous and compact, excluding alginate and water from the feed sources. However, the insect may not be able to find the right way to feed, resulting in random feed acquisition instead of the regular gain that occurs in living prey attacks.

Furthermore, the alginate matrix exhibits numerous water domains (blue in Fig 7). Cryo-SEM imaging reveals water domains within the alginate matrix that can serve as a water reservoir for the insect. In insect-rearing, water is typically provided alongside diets to allow insects to quench their thirst. Microcapsules integrate the diet into a central core, preserving its texture and protecting it from both biotic and abiotic environmental degradation, while also providing multiple sources of water.

However, evidence suggests a significant lateral displacement of the core within microcapsules, resulting in uneven and unpredictable variations in wall thickness from the perspective of *Z. renardii*. A chunky wall in the microcapsules prevents access to the diet core of *Z. renardii*, making the feeding unpredictable and inducing stress and starvation in the predator. The attempts of *Z. renardii* to feed on microcapsules do not unfold the urosternites, demonstrating the predator’s inability to access its feed portion regularly.

However, we were unable to conduct feeding experiments because *Z. renardii* accepts both the offered devices, feeding randomly and unpredictably depending on the size of the microbead diet domains or the microcapsule’s wall thickness.

Finally, microbeads and microcapsules prevented proper foraging until engorgement, as evidenced by the non-unfolding of the abdominal fold. Future studies will focus on centralizing the core for microcapsules, obtaining larger cores if we proceed with the use of microbeads, heating the artifact production pipeline, and possibly replacing the gelling agent.

## 4. Conclusions

We suggest continuing the experience with the use of alginate microbead or microcapsule devices obtained by prilling to offer diet and water portions for *Z. renardii* under mass rearing conditions. The prilling/vibration technique offers interesting and promising multicore microbeads and monocore microcapsules with controlled size and composition.

The cross-section of multicore microbead particles (F2_bis_ and F4) revealed a peculiar distribution of several domains, creating a dispersed and diverse multicore structure. F2_bis_ enhanced insect acceptance due to the oligidic base’s surface coating. However, the matrix’s air bubbles and water domains still pose challenges in optimizing the microbeads’ structure.

Monocore microcapsules (F8) would better fit the feeding requirements for *Z. renardii*. However, their internal structure showed a side shift of the core, likely due to density differences between the oligidic base and the alginate shell. Additionally, microcapsules provide an extra source of water, which would be essential for insect rearing. We suggest focusing on new technical parameters to achieve an entirely centered large diet core and a consequent predictable and standard thin layer of alginate wall, thereby optimizing microcapsule prilling.

Our experience suggests that microbeads and microcapsules can effectively deliver artificial diets to *Z. renardii*, minimizing contamination risks and ensuring controlled nutrient intake. However, the observed artifacts (microbeads and microcapsules) do not reflect an optimal configuration for the predator to feed on. For adequate feeding by *Z. renardii,* the diet should be in a single core or a few large cores, in line with the predator’s feeding habits. We must improve formulation and device parameters by warming the whole pipeline, or changing the gelling agent, i.e., adding gelatin to enhance microparticle homogeneity and stability, while reducing the core’s lateralization and wall thickness, and evaluating their effects on the predator’s acceptance. The microparticles could facilitate additional research on the automation of mass rearing of *Z. renardii*, which can serve for augmentative biocontrol.
